# Supplementary material for: Parents’ and Children’s Experiences with a Coordinating Professional in Integrated Care for Childhood Overweight and Obesity—A Novel Dutch Approach
Source: Int J Environ Res Public Health. 2022 May 10;19(10):5797. doi: 10.3390/ijerph19105797 (PMC9141245; doi:10.3390/ijerph19105797)
Supplement: Supplementary file 1 [file ijerph-19-05797-s001.zip › ijerph-1662174-supplementary.pdf]

Interview protocol evaluation integrated approach childhood overweight and obesity
Parent & child (version 8, 25-11-2019)

Thank you for taking part in this interview. We are [name] and [name], we are researchers from the university in Tilburg and the GGD in Den Bosch. We are curious about your opinion. We would like to hear it if you are satisfied or not. We are going to ask you questions and if you want you can draw something or play a short game. All the answers to our questions are good. There are no wrong answers. We'll start together and then split up, if that's okay with you.

The conversation will take about 1 hour, depending on how much you have to say. We will record the interview so we can transcribe it later. Your answers will be treated confidentially, we will not include any names with the answers.

<u>Interview questions</u>

<u>General</u>

1. Can you introduce yourselves? (possibly first give the example myself)

Ask for occupation parents.

2. What does your family look like?

Possibly to the child: can you draw who lives in your house?

Recently you have been in contact with the youth health care nurse or youth health care doctor from the GGD at the health care centre.

3. With whom do you have/have you had contact?

4. How often do you have/have you had contact?

5. Was this at the GGD or at home?

6. What did you discuss?

7. How did you get into action?

8. What did you think of the supervision by the youth health care nurse/youth health care doctor?

9. Did you feel supported?

10. Did the counselling/guidance help you further?

We would now like to ask the child a number of questions and a number of questions to the parent(s). Do you think it's okay to split up for a moment? Then we will end up together again.

Children

We would like to ask you a few questions about how you are doing.

[Icebreaker: game with questions about what you prefer]

1. Do you know why you are sitting here?

No; explanation integrated approach: you and your parents have been supported to live healthier. We would like to know how you felt about the support].

2. Did a youth health care nurse or youth health care doctor of the GGD (from the health care centre) talk to you about how you are doing? Who was that? How was that? What do you think of him/her? (Show pictures of the youth health care nurses)

3. How does he/she help you and your parents?

4. What are the conversations with the youth health care nurse/youth health care doctor about? What topics do you talk about? (possibly playing with playmobile dolls)

5. Has the youth health care nurse/youth health care doctor listened carefully to what you think is important?

6. Do you think he/she helped you well?

7. What grade would you give the youth health care nurse? (0-10)

8. Are you seeing other doctors or caregivers? Or do they come to your home?

What do you think about this? Do they help you? What grade would you give these people? (0-10)

9. What do you think about paying attention to healthy weight at school and in the district/sports club?

10. What helps you best?

11. What could be better?

12. Is there anything else you'd like to tell me?

Thanks for your help! Because you helped us, you can choose something to play with. You can do something for yourself now, until the conversation with your father/mother is also finished. Then we will talk together for a while.

Parents

General

1. When did overweight/obesity first come up for debate? Who brought it up?

What did you think of this?

2. What support/care is your family receiving right now? And in recent years?

3. What worked well for you as a family?

4. What could be better?

5. What grade do you give the guidance of the youth health care nurse of the GGD (the health care centre)? (0-10)

6. Have you ever thought about quitting counseling? Why did you continue?

Integrated approach childhood overweight and obesity

7. Have you heard of the integrated approach for childhood overweight and obesity in 's-Hertogenbosch?

If so, can you tell us what it is?

If not: we will briefly explain what is meant by the integrated approach.

In 's-Hertogenbosch professionals work together. The youth health care nurse talks about the weight of children with parents and determines together with parents what is needed. If necessary, she asks others to help. For example, a dietician, a social worker or a pediatrician. Also at schools and in the neighborhood, there is extra attention for physical activity and drinking water.

8. Have you noticed anything about this?

9. What do you think of the integrated approach for childhood overweight and obesity?

10. If you have to give the integrated approach in 's-Hertogenbosch as a whole a grade, what would it be? Can you explain this grade? (0-10)

Competences of the youth health care nurse/central care provider

11. We have a number of statements about the youth health care nurse. Can you indicate whether you agree or disagree?

	Agree	Disagree	Don't know (don't give this option immediatel y)
1. The youth health care nurse knows our past.			
2. The youth health care nurse discusses how I can involve friends and family in achieving our goals.			
3. I feel that I can tell everything in the conversation with the youth health care nurse. Even things that are not about weight and food.			
4. The youth health care nurse asked a broad set of questions and discussed what could be the causes of obesity in our case (a broad anamnesis was taken).			
5. The youth health care nurse discusses what makes a healthy lifestyle more difficult for us.			
6. We decide on our own goals/what we want to work on.			
7. Together with us, the youth health care nurse makes an action plan.			
8. The youth health care nurse is coaching us.			
9. The general practitioner knows that the youth health care nurse is there to support us.			

12. We have a number of statements about the work of the youth health care nurse.

You can answer with: never, almost never, sometimes, most of the time, always (scale where they impose the statement; questioning on any obvious answers)

	Never	Almost never	Some times	Most of the time	Always
1. The youth health care nurse involves the child(ren) in the conversations					
2. The youth health care nurse discusses how we can live a healthier live					
3. I understand what the youth health care nurse is telling us					
4. I have the possibility to do what the youth health care nurse advises us to do					
5. The youth health care nurse cooperates with others when necessary					
6. I think the youth health care nurse had enough time for us					

Referrals

13. Has the youth health care nurse or doctor referred you? To whom?

14. How did the referral go?

15. What did it yield?

16. Was there cooperation between the youth health care nurse and the person to whom you were referred?

If so, how did that collaboration go?

Final questions parent + child

1. Together with you, we would like to write a short letter to the youth health care nurse or the person with whom you have had the most contact. What would you like to say to her/him?

Dear ....

What I would like to say to you is:

Top:

Tip:

2. Would you like to say something about the integrated approach for childhood overweight and obesity?

3. What do you think of this interview?

Thank you for your time and sharing your experiences. If you would like to tell me something after this conversation, you can always e-mail me. If I have forgotten something, can I call/mail again?

Once we have finished the interviews we will start to work out the details and write a report. Would you then like to receive a summary of the research?

We are trying to get points for improvement from the interviews for the work of the youth health care nurse and the integrated approach for childhood overweight and obesity. We want to formulate recommendations for, for example, the GGD, the municipality and the Jeroen Bosch Hospital.

We would like to discuss these recommendations with parents; after all, they are about you. Would you like to meet with a group of parents after all the interviews to discuss the tips for improvement?
